# Association of pregnancy complications and postpartum maternal leukocyte telomeres in two diverse cohorts: a nested case-control study

**DOI:** 10.1186/s12884-024-06688-5

**Published:** 2024-07-20

**Authors:** Danielle M. Panelli, Xiaobin Wang, Jonathan Mayo, Ronald J. Wong, Xiumei Hong, Martin Becker, Nima Aghaeepour, Maurice L. Druzin, Barry S. Zuckerman, David K. Stevenson, Gary M. Shaw DrPH, Katherine Bianco

**Affiliations:** 1 Division of Maternal-Fetal Medicine and Obstetrics, Department of Obstetrics and Gynecology, Stanford University School of Medicine, Center for Academic Medicine, Obstetrics and Gynecology, MC 5317, 453 Quarry Road, Stanford, CA 94304 USA; 2 Center on the Early Life Origins of Disease, Departments of Population, Family and Reproductive Health, Johns Hopkins Bloomberg School of Public Health, Baltimore, MD USA; 3 Department of Pediatrics, Stanford University School of Medicine, Stanford, CA USA; 4 Department of Anesthesiology, Perioperative, and Pain Medicine, Stanford University School of Medicine, Stanford, CA USA; 5 Department of Computer Science and Electrical Engineering, University of Rostock, Rostock, Germany; 6 Department of Biomedical Data Science, Stanford University, Stanford, CA USA; 7 Department of Pediatrics, Boston Medical Center, Boston, MA USA

**Keywords:** Pregnancy, Preeclampsia, Preterm birth, Telomeres, Cellular aging, Disparity, Stress, Hypertensive disorders

## Abstract

**Background:**

Biologic strain such as oxidative stress has been associated with short leukocyte telomere length (LTL), as well as with preeclampsia and spontaneous preterm birth, yet little is known about their relationships with each other. We investigated associations of postpartum maternal LTL with preeclampsia and spontaneous preterm birth.

**Methods:**

This pilot nested case control study included independent cohorts of pregnant people with singleton gestations from two academic institutions: Cohort 1 (hereafter referred to as Suburban) were enrolled prior to 20 weeks’ gestation between 2012 and 2018; and Cohort 2 (hereafter referred to as Urban) were enrolled at delivery between 2000 and 2012. Spontaneous preterm birth or preeclampsia were the selected pregnancy complications and served as cases. Cases were compared with controls from each study cohort of uncomplicated term births. Blood was collected between postpartum day 1 and up to 6 months postpartum and samples were frozen, then simultaneously thawed for analysis. Postpartum LTL was the primary outcome, measured using quantitative polymerase chain reaction (PCR) and compared using linear multivariable regression models adjusting for maternal age. Secondary analyses were done stratified by mode of delivery and self-reported level of stress during pregnancy.

**Results:**

156 people were included; 66 from the Suburban Cohort and 90 from the Urban Cohort. The Suburban Cohort was predominantly White, Hispanic, higher income and the Urban Cohort was predominantly Black, Haitian, and lower income. We found a trend towards shorter LTLs among people with preeclampsia in the Urban Cohort (6517 versus 6913 bp, *p* = 0.07), but not in the Suburban Cohort. There were no significant differences in LTLs among people with spontaneous preterm birth compared to term controls in the Suburban Cohort (6044 versus 6144 bp, *p* = 0.64) or in the Urban Cohort (6717 versus 6913, *p* = 0.37). No differences were noted by mode of delivery. When stratifying by stress levels in the Urban Cohort, preeclampsia was associated with shorter postpartum LTLs in people with moderate stress levels (*p* = 0.02).

**Conclusion:**

Our exploratory results compare postpartum maternal LTLs between cases with preeclampsia or spontaneous preterm birth and controls in two distinct cohorts. These pilot data contribute to emerging literature on LTLs in pregnancy.

**Supplementary Information:**

The online version contains supplementary material available at 10.1186/s12884-024-06688-5.

## Introduction

Pregnancy complications such as preeclampsia or spontaneous preterm birth have been linked to increases in future morbidity in the birthing parent across their lifespan [1–3]. This may be because these pregnancy complications are related to mechanisms that lay the biologic foundation for future age-related morbidities, such as development of ischemic heart disease [3]. In fact, preeclampsia and spontaneous preterm birth have been associated with biologic changes that can persist beyond pregnancy, which may directly contribute to their known long-term disease risks [4, 5]. However, the biologic changes that have been previously examined are frequently heterogeneous or multifactorial, highlighting the need for research on biomarkers that better reflect the cumulative impact of various stress-related pathways.

One biomarker with potential as a surrogate for accumulated biologic stress is leukocyte telomere length (LTL). Telomeres are repetitive non-coding DNA sequences that protect the ends of linear chromosomes from degradation over time with cell division [6, 7]. Short LTL have been found to be both a consequence and predictor of morbidity; for example, inflammation and oxidative stress shorten LTL, and short LTLs are associated with increased future morbidity and early mortality [8, 9]. Much of the literature in non-pregnant adults focuses on LTL for several reasons, such as: (1) the fact that telomeres are distinct between tissues yet correlate by individual; (2) the relative ease of sampling; and (3) the fact that inflammation promotes white blood cell turnover as manifested by short LTL [10].

**Fig. 1:**
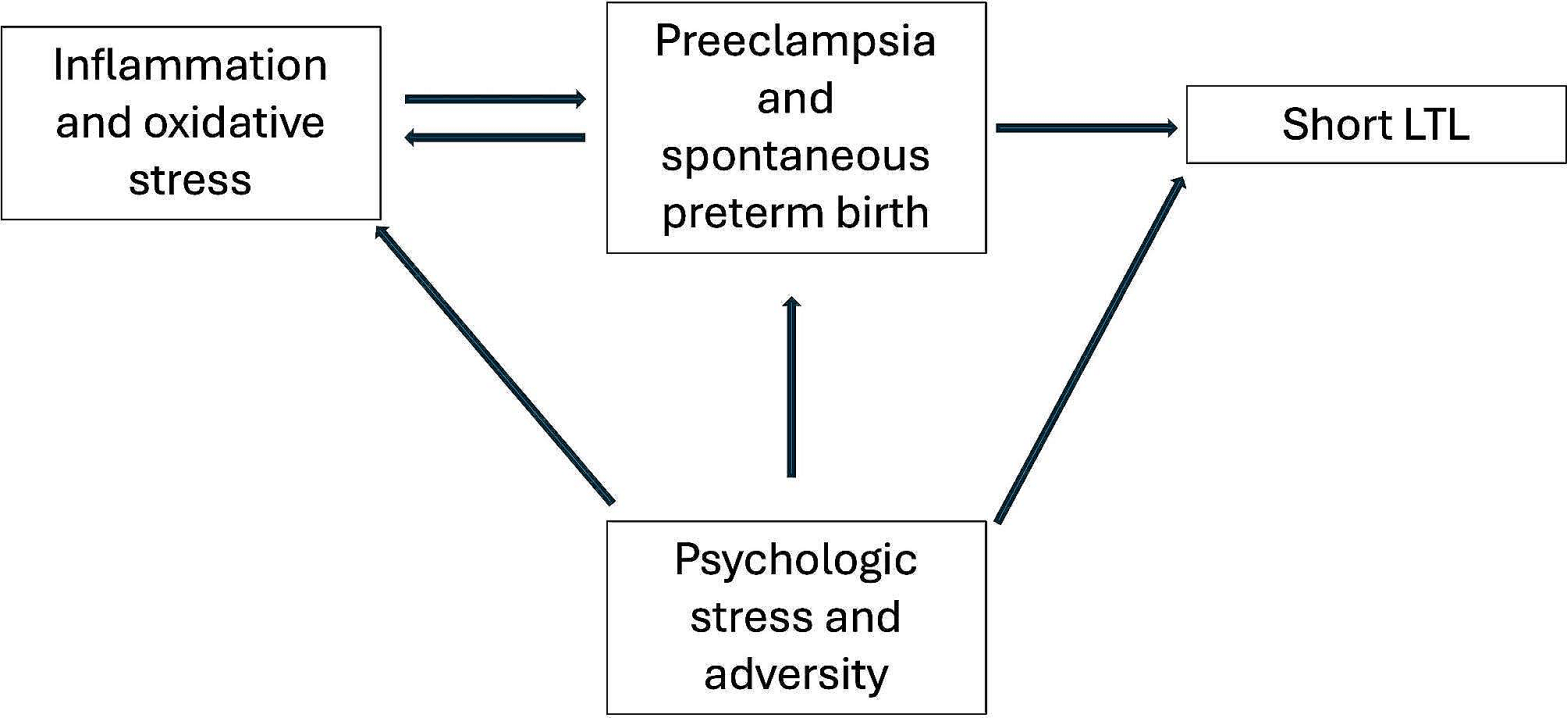
Proposed causal pathway

While data are limited on LTL dynamics in pregnancy, emerging data suggest the potential relationship between LTLs, socioeconomic stress, adversity, and complications such as spontaneous preterm birth [7, 11]. However, little is known about how pregnancy complications affect LTL, or whether LTL is predictive of pregnancy complications. The latter question has been explored by our group as well as others, and is an area of ongoing investigation [12–14]. The objective of this exploratory study was to examine the former question of whether potential immune-related pregnancy complications (preterm birth and preeclampsia) [15–17] affect postpartum maternal LTL. In the present study, we hypothesized that postpartum maternal LTLs would be shorter in pregnancies complicated by preeclampsia or spontaneous preterm birth, compared to pregnancies with neither of these complications (Fig. 1).

## Materials and methods

### Study design

This pilot nested case control study investigated two independent cohorts to explore a potential association of two selected pregnancy complications – preeclampsia and spontaneous preterm birth – with postpartum maternal LTL. The Suburban Cohort comprised pregnant people with singleton pregnancies enrolled prior to 20 weeks’ gestation in a longitudinal study conducted by a Prematurity Research Center at a West Coast academic university from 2012 to 2018. The Urban Cohort comprised pregnant people with singleton pregnancies enrolled at delivery in a longitudinal study conducted by a prospective birth cohort at an East Coast academic university between 2000 and 2012. Enrollment procedures for both cohorts have previously been described [18–21]. In both study cohorts, serial blood samples were obtained from participants and frozen (at -80 °C for the Suburban Cohort and − 20 °C for the Urban Cohort). Participants from both study cohorts were eligible for inclusion in this study if they had blood samples and medical records from the delivery universities (IRB Protocol Number 21,956, Protocol Number 3966/CR1004). Written informed consent for participation future research was obtained from all who enrolled in both studies.

Cases were defined as those individuals who had spontaneous preterm birth < 37 weeks or preeclampsia (with or without severe features) meeting inclusion criteria identified in both cohorts. Determination of spontaneous preterm birth or preeclampsia was made directly from review of medical records. Preeclampsia was only diagnosed if present up to the point of the delivery hospitalization; additional records beyond the immediate hospitalization postpartum period were not evaluated for this. Any participant who experienced both preeclampsia and spontaneous preterm birth was classified as a preeclampsia case and not a preterm birth case, as the preeclampsia preceded the preterm birth. Medically-indicated preterm births were not included as cases. As controls, we randomly selected individuals from each cohort who did not develop preeclampsia prior to blood sample collection and delivered at or beyond 37 weeks’ gestation. Of note, we did not exclude individuals who experienced preterm labor yet delivered at or beyond 37 weeks from the control group.

The primary outcome was postpartum maternal LTL, obtained from the blood draw most proximal to the time of delivery for cases and controls in both cohorts. In the Suburban Cohort, this corresponded with a blood draw done from postpartum day 1 up to 6 weeks’ postpartum. BD Vacutainer CPT Cell Preparation Tubes with Sodium Citrate were used for collection. In the Urban Cohort, this corresponded with a blood draw done within 24 to 72 h after delivery. Sample processing and storage procedures for both cohorts have been previously described [19].

In the Suburban Cohort, samples were thawed at room temperature and genomic DNA was extracted from whole blood using the QIAamp protocol for cultured cells [22]. In the Urban Cohort, genomic DNA was isolated from EDTA-treated peripheral white blood cells and DNA was extracted in accordance with standard protocols as previously described [23, 24]. The concentration and purity were determined using a Quant-iT™ Broad-range dsDNA Assay Kit on a SpectraMax M2 microplate reader.

After DNA extraction, LTL was measured in all samples in accordance with the methods previously described by Cawthon et al. at the same laboratory using the same reagents [19, 25, 26]. Using this protocol, telomeric “T” and single copy gene “S” – derived from human beta globin – lengths were calculated from each sample as well as from standard reference DNA samples via quantitative polymerase chain reaction (qPCR). Average “T” and “S” concentrations were used to calculate T/S ratios twice per sample, and for any that differed by greater than 7% the T/S ratio was measured a third time and the two closest values were averaged and reported as a single T/S ratio per sample. Adjustment factors were obtained using control samples to account for potential batch effect. These adjusted T/S ratios were converted to basepairs (bp) using a standard conversion developed in the same laboratory [bp = 3274 + 2413*(adjusted T/S)] [27, 28]. We chose to report LTL in base pairs rather than T/S ratios for interpretability with other literature.

Secondary analyses were conducted stratifying results by stress levels. Stress levels were ascertained from each cohort during enrollment. In the Suburban Cohort, participants were given questionnaires assessing psychosocial stressors at the time of enrollment during the first trimester of pregnancy. Stressors assessed on these questionnaires included those related to perceived risk of pregnancy complications, stress level, and sleep quality as detailed in previously published work [18, 19]. Questions regarding perceived risk of birth complications were asked directly to the participants. The DQAQ-SPF questionnaire assessed the remaining stressors, using either an eight point scale for low/moderate/high questions or yes/no for binary questions about specific life experiences [18]. In the Urban Cohort, questionnaire-based interviews were conducted after enrollment in the immediate postpartum period to assess stress levels during pregnancy. The question “How would you characterize the amount of stress during your life in this pregnancy” and its three level response (low, moderate, or high) assessed stress in the Urban Cohort as detailed in prior work [20].

Due to differences in sample collection protocols and timing between the two cohorts, analyses were conducted in parallel separately for each cohort. First, demographic and clinical variables (maternal age, self-reported race and ethnicity as social constructs, body mass index (BMI) at enrollment, parity, mode of delivery, and gestational age at birth) obtained from medical records were described across cases and controls. Next, the relationship between LTL and cases with spontaneous PTB or preeclampsia versus controls was assessed in a generalized linear model stratified by Suburban/Rural cohort. These were initially done as crude models followed by multivariable models including maternal age as a covariate. Maternal age was selected a priori as the strongest potential confounder due to known associations with preterm birth [29], preeclampsia [30], and short LTL [31]. We then performed a sensitivity analysis replicating the multivariable models stratifying by mode of delivery in the event that postpartum white blood cell differential changes might affect results. Secondarily, we explored associations between maternal perception of pregnancy-associated stress and LTL using responses from stress questionnaires in each cohort. In the Suburban Cohort, we conducted univariate analysis to investigate the relation of individual survey responses and LTLs. In the Urban Cohort, we replicated the primary linear regression models stratified by low, moderate, and high stress levels. Sample sizes were selected based on sample availability for this pilot analysis.

## Results

**Fig. 2:**
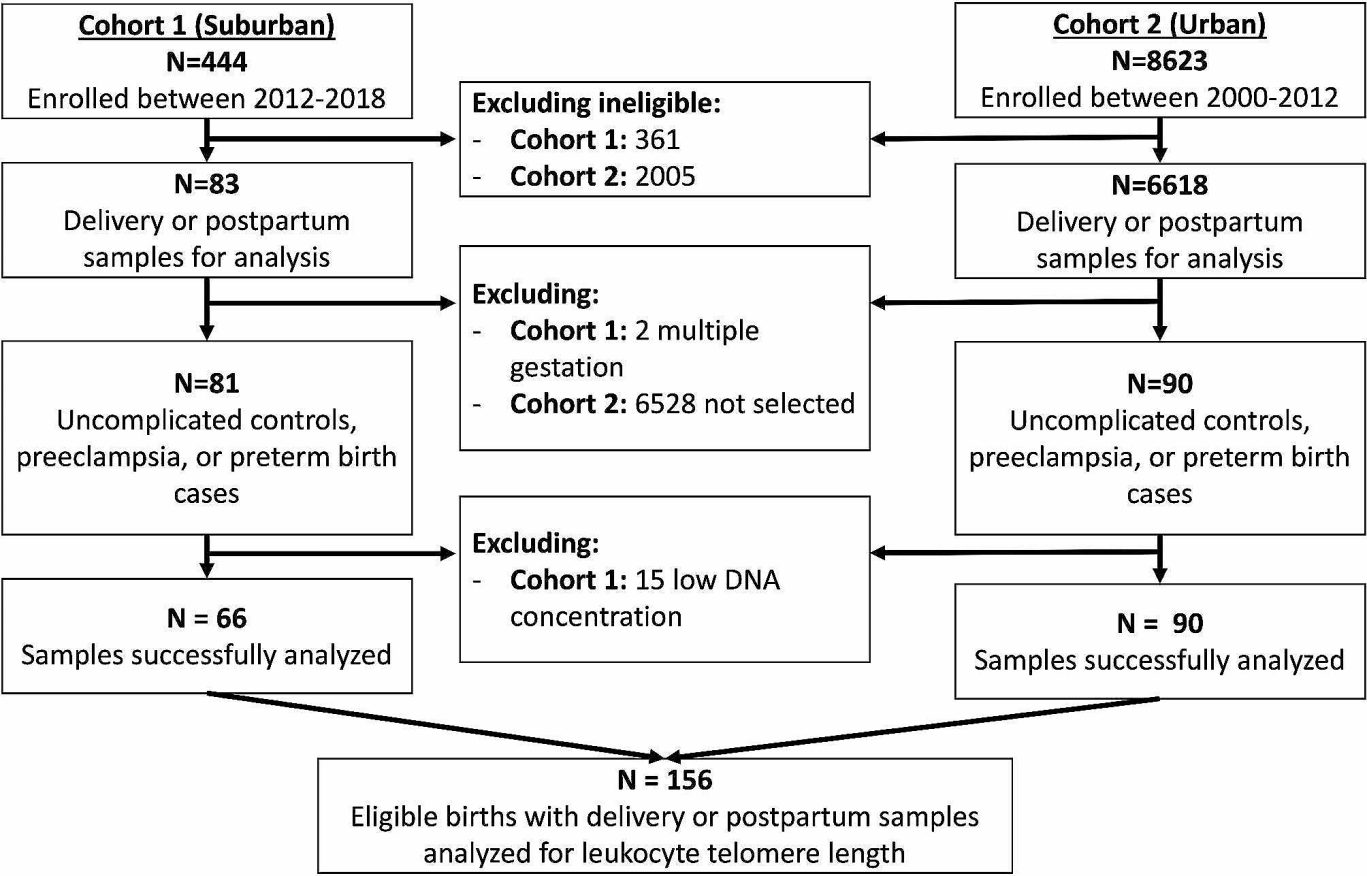
Consort diagram

Of 171 eligible participants, 156 (91.2%) had blood samples with adequate DNA concentrations for LTL analysis; 66 from the Suburban Cohort and 90 from the Urban Cohort (Fig. 2).

Table 1 shows the demographics and pregnancy characteristics for cases and controls across the two cohorts. The Suburban Cohort was predominantly suburban of Non-Hispanic White, Hispanic, or Asian race/ethnicity and the Urban Cohort was predominantly urban of Non-Hispanic Black or Haitian race/ethnicity. In the Suburban Cohort, of the 64 people in whom education level was known, 45 (70.3%) had an undergraduate degree or above. In the Urban Cohort, of the 89 people in whom education level was known, only 12 (13.5%) had an undergraduate degree or above. Additional description of the Urban Cohort’s sociodemographic profile has been previously published [20]. Mean (standard deviation) gestational age at enrollment in the Suburban Cohort was 11.0 (2.6) weeks for preeclampsia, 12.3 (6.7) weeks for spontaneous preterm birth, and 10.1 (1.8) weeks for control groups. Enrollment in the Urban Cohort occurred on postpartum day 0–1 for all participants. One-third of blood samples in the Suburban Cohort were drawn during the first postpartum week compared with 100% of samples in the Urban Cohort. Among the Suburban Cohort, mean LTL did not differ between the 20 people who had samples drawn postpartum week 1 and the 46 people who had it drawn later (mean [standard deviation] 6179 versus 6086, *p* = 0.59). Of note, 3 people in the Suburban Cohort (4.5%) and 11 people in the Urban Cohort (12.2%) received diagnoses of either chorioamnionitis or endometritis.

**Table 1:** Demographics and pregnancy characteristics of two diverse cohorts of pregnancies complicated by preeclampsia, spontaneous preterm birth, or controls with neither (*N* = 156)

	Overall^a^	Suburban Cohort^a^ *N* = 66			Urban Cohort^a^ *N* = 90		
	*N* = 156	Control *N* = 33	Pre-eclampsia *N* = 21	Preterm Birth *N* = 12	Control *N* = 30	Pre-eclampsia *N* = 30	Preterm Birth *N* = 30
Median (IQR) maternal age (years)	30.5 (7.5)	30 (5)	36 (6)	33 (3)	29 (9)	29 (10)	32 (8)
Race/ethnicity^c^							
American Indian or Asian	7 (4.5)	1 (3.0)	7 (33.3)	1 (8.3)	0	0	0
Haitian	28 (18.0)	0	0	0	8 (26.7)	12 (40.0)	8 (26.7)
Hispanic	19 (12.2)	9 (27.3)	8 (38.1)	2 (16.7)	0	0	0
Non-Hispanic Black	63 (40.4)	0	1 (4.8)	0	22 (73.3)	18 (60.0)	22 (73.3)
Non-Hispanic White	32 (20.5)	21 (63.6)	5 (23.8)	6 (50.0)	0	0	0
Multiple or Other	5 (3.2)	1 (6.1)	0	3 (25.0)	0	0	0
Median (IQR) body-mass index (kg/m^2^) at enrollment	25.8 (9.7)	23.1 (6.7)	26.6 (11.2)	25.0 (7.2)	24.6 (6.2)	30.6 (6.3)	27.1 (11.2)
Nulliparous	61 (39.1)	14 (42.4)	6 (28.6)	2 (16.7)	10 (33.3)	18 (60.0)	11 (36.7)
Current or former smoker	20 (12.8)	1 (3.0)	2 (9.5)	2 (16.7)	3 (10.0)	4 (13.3)	8 (26.7)
Cesarean birth	58 (37.2)	8 (24.2)	12 (57.1)	8 (66.7)	9 (30.0)	11 (36.7)	10 (33.3)
Median (IQR) gestational age of birth (completed weeks)	37.0 (4.0)	39.0 (1.0)	37.0 (3.0)	36.0 (3.0)	39.5 (2)	36.0 (3)	34.0 (4)
Timing of blood draw							
First week	110 (70.5)	10 (30.3)	6 (28.6)	4 (33.3)	30 (100.0)	30 (100.0)	30 (100.0)
Beyond first week	46 (29.5)	23 (69.7)	15 (71.4)	8 (66.7)	0	0	0

^a^ Continuous variables shown as median (interquartile range) and categorical variables as N (column %)

Postpartum maternal LTL was compared between cases and controls in both cohorts as shown in Table 2. In the Suburban Cohort, after adjusting for maternal age, no statistically significant differences were observed in LTL between people with either preeclampsia or spontaneous preterm birth versus controls. In the Urban Cohort, a non-significant trend towards shorter LTLs was observed in people with preeclampsia versus controls, but no statistically significant differences were observed in people with spontaneous preterm birth versus controls. Similar findings were seen when stratifying analyses by mode of delivery (Supplemental Table 1).

**Table 2:** Postpartum maternal leukocyte telomere lengths (LTL) compared between people with pregnancies complicated by preeclampsia, spontaneous preterm birth, or neither in two diverse cohorts (*N* = 156)

	Crude LTL^a^ (mean, 95% CI)	LTL adjusted for maternal age (mean, 95% CI)	Adjusted beta coefficient (95% CI)^b^	*P*-value ^b^
**Suburban Cohort**				
Controls	6118 (5902, 6335)	6144 (5920, 6367)	Reference	-
Preeclampsia	6143 (5872, 6414)	6107 (5824, 6390)	-37 (-412, 338)	0.85
Spontaneous preterm birth	6050 (5692, 6409)	6044 (5687, 6401)	-100 (-524, 343)	0.64
**Urban Cohort**				
Controls	6916 (6608, 7223)	6913 (6611, 7215)	Reference	-
Preeclampsia	6539 (6231, 6846)	6517 (6214, 6820)	-396 (-824, 32)	0.07
Spontaneous preterm birth	6692 (6385, 7000)	6717 (6414, 7020)	-196 (-624, 233)	0.37

^a^ LTL shown in basepairs, rounded to nearest whole number

^b^ Linear regression model adjusting for maternal age. Beta rounded to nearest whole number

Separate secondary analyses were then conducted in each cohort using available stress constructs. In the Urban Cohort, among people with moderate stress levels, only preeclampsia was associated with shorter postpartum LTLs (adjusted beta − 601, 95% confidence interval [CI] -1105 to -98, *p* = 0.02, Table 3). However, significant associations between preeclampsia and short LTL were not seen among those with low or high stress levels. In the Suburban Cohort, due to the variability in stress questions, individual associations between the stress-related questions and short LTLs were examined. Short LTLs were most associated with poor sleep quality (*p* = 0.003) and perceived risk of birth complications (*p* = 0.02, Table 4, Supplemental Table 2).

**Table 3:** Association between preeclampsia and postpartum leukocyte telomere length stratified by self-reported pregnancy stress level in the Urban Cohort (*N* = 60)

Stress level	Preeclampsia *N* = 30 *N* (%)	Postpartum Leukocyte Telomere Length Adjusted Beta^a^ (95% CI)	*p*-value
Low (*N* = 22)	9 (30.0)	-451 (-1146, 243)	0.20
Moderate (*N* = 30)	18 (60.0)	-601 (-1105, -98)	0.02
High (*N* = 8)	3 (10.0)	-454 (-1926, 1019)	0.55

^a^Linear regression adjusting for maternal age

**Table 4:** Selected suburban cohort stress survey questions and association with postpartum leukocyte telomere length

Survey question	*P*-value
Generally, how good is your quality of sleep the night before working days?	0.002978
How happy have you been for most of the time during the past 3 years?	0.014117
How sad have you been for most of the time during the past 3 years?	0.016039
How would you say that your degree of risk of birth complications is compared to most other pregnant women your age? *	0.019588
How intense is your pain?	0.030779
How stressful was your life between the ages of 31 and 40?	0.031593
Did you have another stressful experience?	0.032865
Were your parents divorced?	0.036113
How often do you experience pain?	0.042645
Did a parent lose a job?	0.058179
How would you say that your degree of risk of pregnancy complications is compared to most other pregnant women your age? *	0.059284
How tired/fatigued have you been for most of the time during the past 3 years?	0.069386
How depressed have you been for most of the time during the past 3 years?	0.086059
Number of individuals you could absolutely count on in times of trouble	0.089142
How anxious would you say you generally are as you go about your day-to-day activities?	0.103919
How stressful has the past one year been for you?	0.192164
How angry have you been for most of the time during the past month?	0.197865
Do you engage in any vigorous physical exercise currently?	0.21
Marital status	0.218714
How lonely have you been for most of the time during the past month?	0.238227
How pessimistic are you?	0.261541
How would you say that your degree of risk of a baby with a birth defect is compared to most other pregnant women your age? *	0.268311

*paraphrased

## Comment

### Principal findings

In this exploratory nested case control study conducted in parallel among two diverse cohorts, neither spontaneous preterm birth nor preeclampsia was observed to be associated with postpartum LTLs. When stratifying by stress levels in the Urban Cohort – which was a predominantly Black or Haitian, inner-city population – preeclampsia was significantly associated with shorter LTLs in people with moderate pregnancy stress.

### Results in the context of what is known

Given research suggesting that biologic origins of spontaneous preterm birth and preeclampsia may be inflammatory and begin prior to 20 weeks’ gestation, we had hypothesized that cumulative biologic stress-related changes might be detectable in postpartum maternal LTL measurements [32, 33]. Further, some of these biologic origins may share common pathways (i.e. via immune or metabolomic alterations) to both preeclampsia and preterm birth, though this is an area of active investigation [14, 16]. Though our results did not support this hypothesis, it is possible that the immediate postpartum period is too soon to see such effects on LTL from pregnancy complications. Measuring LTL further out from the index delivery may reveal pathologic changes not yet identifiable in the immediate postpartum period. As we have previously shown that LTL doesn’t dramatically change during healthy pregnancies, it is also possible that postpartum LTL is driven by a person’s LTL prior to or early in pregnancy which may separately predispose them to complications [12, 19]. This could be interpreted as chronic life stress prior to and during pregnancy driving shorter LTL. Alternatively, it may also be that the immunosuppressed state of pregnancy mitigates some of the LTL changes that would otherwise be expected in response to inflammation or stress outside of pregnancy.

Alternatively, given the exploratory nature of our small pilot study, it may be that we were underpowered to detect significant short-term associations between LTLs and the selected pregnancy complications. For example, results from our prior work linking shorter first trimester LTL with earlier gestational age at delivery did not translate to an increase in spontaneous preterm birth in this study which would have been expected. The trends we identified between preeclampsia and LTL – specifically in our predominantly Urban Cohort with moderate stress levels – may indicate a signal worth investigating on a larger scale in this population in particular [20]. While limited by the type of stress assessment that was done in the Urban Cohort, those who endorsed “moderate” stress levels may be skewed to more severe types of stress compared to that experienced by the Suburban Cohort. Given their unique experiences and potential exposure to structural racism over time, it may be that they are particularly susceptible to the biologic stress imparted by preeclampsia [21]. These results complement findings from a recent study by Lueth et al. [34], where a high allostatic stress biomarker load (e.g. cholesterol, C-reactive protein, and triglycerides) was found to be a partial mediator of the association between race or ethnicity and hypertensive disorders of pregnancy. Taken together, these findings highlight the importance of further characterizing the relationship between preeclampsia and biomarkers of stress across different populations over time as associations may vary based on lived experiences and social factors.

### Clinical implications

Preeclampsia, spontaneous preterm birth, and short LTLs have all separately been linked to increased future age-related health risks such as heart failure, coronary artery disease, stroke, and myocardial infarction [1–3, 35]. These risks are heightened among minority populations, particularly those who have suffered from chronic stress due to longstanding societal and structural disparities [36, 37]. Given our finding of shorter LTL in postpartum cases of preeclampsia with moderate stress levels in the Urban Cohort, there may be a window of opportunity in the immediate postpartum period to intervene before biologic strain becomes irreversible. This underscores the importance of future work enhancing postpartum healthcare and optimizing community resources, particularly for those with pregnancy complications, to mitigate long term LTL shortening and its potential consequences.

### Research implications

In addition to prompting the question of how preeclampsia or spontaneous preterm birth might affect telomeres measured more distally from the index pregnancy, our results have other research implications. LTLs have recently been highlighted for their potential role in mediating racial disparities in preterm birth produced by exposure to structural and social factors. Though commenting on this is beyond the scope of our present study, our results highlight the importance of considering how psychosocial stressors could differentially affect populations on a biologic level. This complements prior work establishing a link between psychosocial stressors and pregnancy complications [38–40]. In addition to its potential in evaluating disparities as both social and biological constructs, telomere length should be further explored as a biological surrogate for future health problems given associations with psychosocial stress, trauma, and adversity outside of pregnancy [9, 41, 42]. Additional investigation is warranted into why our results in the Urban Cohort showed significant telomere shortening with moderate but not high stress after preeclampsia. This may be related to sample size, or to a more nuanced relationship. It is important to note that even “moderate” self-reported stress in the Urban Cohort may be higher than average given social and structural disadvantages. Lastly, since maternal psychosocial stress has been associated with short neonatal LTLs, future research investigating how pregnancy complications such as preeclampsia might affect neonatal telomeres is warranted [28].

### Strengths and limitations

The strengths of our study include the simultaneous investigation of two diverse cohorts and granular, patient-level data. Limitations include those inherent to nested case control biologic study designs across multiple sites. First, different DNA extraction techniques were done in the two cohorts so the LTLs obtained for each cohort should not be compared with one another. Second, we selected LTL as the dependent variable in our models since biologic sampling came chronologically after preterm birth or preeclampsia occurred. LTLs on postpartum day 1 could instead reflect overall telomere length leading up to either pregnancy complication rather than in response to the complications. This limitation could be addressed in future research by sampling telomeres in postpartum people at a longer interval after delivery [19]. Differences in stress questionnaires could potentially have contributed to the differing results between the cohorts. To minimize bias from varied storage time and conditions between the two cohorts, we conducted the analyses in parallel rather than pooled, but there may be remaining underlying differences based on sample storage and conditions that remain unaccounted for. Smoking was not considered due to the nature of the study as well as the unreliability of retrospective self-reported smoking status in pregnancy [43]. Lastly, our sample size was limited by availability of biologic samples from the parent cohorts and it is possible that Type 2 error contributed to our findings – particularly for the stress subanalyses. Using the strongest results in the Urban Cohort with preeclampsia versus controls, using a LTL difference of 377 bp, standard deviation of 815 bp, alpha 0.05, 1:1 ratio, and the experimental sample size of 30, our power is estimated to be 42% to reject the null hypothesis. To achieve 80% power, at least 70 participants would be needed per group. Despite this limitation, there is minimal data describing postpartum LTLs and our pilot study results are useful for designing future research in this area. This is particularly important given the potential for integration with other stress-related phenotypes and pregnancy outcomes as next steps in this work [38].

## Conclusions

In this exploratory pilot nested case control study, we did not identify significant associations between preeclampsia or spontaneous preterm birth and maternal LTL in the immediate postpartum period. Preeclampsia was associated with shorter postpartum LTLs when stratifying by stress levels in the cohort with more potential longstanding exposure to chronic stress and transgenerational marginalization and adversity, which may warrant additional investigation in larger, diverse cohorts.

### Electronic supplementary material

Below is the link to the electronic supplementary material.

Supplementary Material 1

## Data Availability

The datasets generated and analyzed in the current study are not publicly available, but may be available from the corresponding author on reasonable request.

## Abbreviations

Bp: Basepairs
CV: Coefficient of variation
LTL: Leukocyte telomere length
GA: Gestational age
PBMCs: Peripheral blood mononuclear cells
T/S ratio: Telomere to single copy gene ratio
