# Supplementary material for: Association of pregnancy complications and postpartum maternal leukocyte telomeres in two diverse cohorts: a nested case-control study
Source: BMC Pregnancy Childbirth. 2024 Jul 20;24:490. doi: 10.1186/s12884-024-06688-5 (PMC11264806; doi:10.1186/s12884-024-06688-5)
Supplement: Supplementary file 1 — Supplementary Material 1 [file 12884_2024_6688_MOESM1_ESM.docx]

**Supplemental Table 1.** **Postpartum maternal leukocyte telomere lengths (LTL) compared between people with pregnancies complicated by preeclampsia, spontaneous preterm birth, or neither in two diverse cohorts, stratified by mode of delivery (N=156).**

| **Cohort** | **Mode of delivery** | **Case versus Control** | **LTL adjusted for maternal age^a^**  **(mean, 95% CI)** | **Adjusted beta coefficient**  **(95% CI)^b^** | **P-value ^b^** |
| --- | --- | --- | --- | --- | --- |
| **Cohort 1** | Vaginal | Controls (N=25) | 6304 (6057,6550) | - | - |
|  |  | Preeclampsia  (N=9) | 6044 (5605, 6484) | -260 (-791, 272) | 0.34 |
|  |  | Spontaneous preterm birth  (N=4) | 6330 (5740, 6920) | 26.4 (-613, 666) | 0.94 |
|  | Cesarean | Controls  (N=8) | 5734 (5329, 6139) | - | - |
|  |  | Preeclampsia  (N=12) | 6088 (5758, 6417) | 354 (-172, 879) | 0.18 |
|  |  | Spontaneous preterm birth  (N=8) | 5910 (5329, 6139) | 176 (-396, 747) | 0.55 |
| **Cohort 2** | Vaginal | Controls  (N=21) | 6888 (6568, 7209) | - | - |
|  |  | Preeclampsia  (N=19) | 6609 (6271, 6947) | -279 (-744, 186) | 0.24 |
|  |  | Spontaneous preterm birth  (N=20) | 6501 (6170, 6833) | -387 (-850, 76) | 0.10 |
|  | Cesarean | Controls  (N=9) | 6976 (6352, 7600) | - | - |
|  |  | Preeclampsia  (N=11) | 6359 (5796, 6922) | -617 (-1460, 225.5) | 0.15 |
|  |  | Spontaneous preterm birth  (N=10) | 7141 (6550, 7731) | 165 (-696, 1025) | 0.71 |

^a^ LTL shown in basepairs, rounded to nearest whole number.

^b^ Linear regression model adjusting for maternal age. Beta rounded to nearest whole number.

**Supplemental Table 2. Suburban Cohort stress survey questions and association with postpartum leukocyte telomere length.**

| **Survey question** | **Test** | **P-value** |
| --- | --- | --- |
| Generally, how good is your quality of sleep the night before working days? | Kendall | 0.002978 |
| How happy have you been for most of the time during the past 3 years? | Kendall | 0.014117 |
| How sad have you been for most of the time during the past 3 years? | Kendall | 0.016039 |
| How would you say that your degree of risk of birth complications is compared to most other pregnant women your age?* | Kendall | 0.019588 |
| How intense is your pain? | Kendall | 0.030779 |
| How stressful was your life between the ages of 31 and 40? | Kendall | 0.031593 |
| Did you have another stressful experience? | Wilcoxon Rank Sum | 0.032865 |
| Were your parents divorced? | Wilcoxon Rank Sum | 0.036113 |
| How often do you experience pain? | Kendall | 0.042645 |
| Did a parent lose a job? | Wilcoxon Rank Sum | 0.058179 |
| How would you say that your degree of risk of pregnancy complications is compared to most other pregnant women your age?* | Wilcoxon Rank Sum | 0.059284 |
| How tired/fatigued have you been for most of the time during the past 3 years? | Kendall | 0.069386 |
| How depressed have you been for most of the time during the past 3 years? | Kendall | 0.086059 |
| Number of individuals you could absolutely count on in times of trouble | Wilcoxon Rank Sum | 0.089142 |
| How anxious would you say you generally are as you go about your day-to-day activities? | Kendall | 0.103919 |
| Have you lost your own home? | Wilcoxon Rank Sum | 0.116313 |
| Stress before kindergarden | kruskal | 0.136571 |
| Death of a loved one | Wilcoxon Rank Sum | 0.142101 |
| Do you use glasses? | kruskal | 0.16731 |
| How stressful has the past one year been for you? | Kendall | 0.192164 |
| How angry have you been for most of the time during the past month? | Kendall | 0.197865 |
| Do you engage in any vigorous physical exercise currently? | Wilcoxon Rank Sum | 0.21 |
| Marital status | Wilcoxon Rank Sum | 0.218714 |
| How lonely have you been for most of the time during the past month? | Kendall | 0.238227 |
| How pessimistic are you? | Kendall | 0.261541 |
| How would you say that your degree of risk of a baby with a birth defect is compared to most other pregnant women your age?* | Kendall | 0.268311 |
| How optimistic are you? | Kendall | 0.279794 |
| Do you engage in any moderate physical exercise currently? | Kendall | 0.28364 |
| Preferred wake time | Kendall | 0.292628 |
| Generally, how good is your quality of sleep the night before weekends and holidays? | Kendall | 0.306872 |
| Do you engage in any vigorous physical activity currently? | Kendall | 0.317311 |
| Please state your approximate number of friends on Facebook | Kendall | 0.322005 |
| How religious are you? | Kendall | 0.414918 |
| How stressful were the first 10 years of your life? | Kendall | 0.416343 |
| How stressful was your life between the ages of 11 and 20? | Kendall | 0.457215 |
| How satisfied have you generally been with your height since your late teenage years | Kendall | 0.504446 |
| How shy are you? | Kendall | 0.586439 |
| Did you experience bullying? | Wilcoxon Rank Sum | 0.59536 |
| How spiritual are you? | Kendall | 0.604994 |
| Have you lost your own job? | Wilcoxon Rank Sum | 0.735853 |
| How stressful have the past 3 years been for you? | Kendall | 0.765693 |
| Have you been divorced? | Wilcoxon Rank Sum | 0.76769 |
| Have you had a major illness? | Wilcoxon Rank Sum | 0.782148 |
| Estimated hours of rest | Kendall | 0.796319 |
| Estimated time preferred to go to bed | Kendall | 0.828621 |
| How many hours of sleep did you get last night? | Kendall | 0.881736 |
| Estimated time to wake | Kendall | 0.882245 |
| Do you presently work outside the home? | Wilcoxon Rank Sum | 0.899939 |
| How healthy do you feel at this moment? | Kendall | 0.902743 |
| How stressful was your life between the ages of 21 and 30? | Kendall | 0.913492 |
| How many hours do you work? | Kendall | 0.940492 |
| How satisfied have you generally been with your body weight since your late teenage years? | Kendall | 0.947314 |
| How healthy have you felt for most of the time during the past 3 years? | Kendall | 0.958358 |
| How supportive has the baby’s father been during the past 1 year? | Kendall | 0.984567 |
| How stressful was your life between the ages of 41 and 50? | Kendall | 1 |
| How satisfied have you generally been with your appearance since your late teenage years? | Kendall | 1 |
| What hand do you write with?* | kruskal | 1 |
| Did your parent lose their home? | Wilcoxon Rank Sum | 1 |
| Any serious accident? | Wilcoxon Rank Sum | 1 |

*paraphrased
